# Supplementary material for: Impact of distance and vertical placement of distal implants on bone mechanics in bar-retained overdenture treatment: A 3D finite element study
Source: PLoS One. 2025 Dec 30;20(12):e0339761. doi: 10.1371/journal.pone.0339761 (PMC12753058; doi:10.1371/journal.pone.0339761)
Supplement: S1 Data — (DOCX) [file pone.0339761.s001.docx]

**All Data Set of the study named**

**PONE-D-25-47053**

**Impact of Distance and Vertical Placement of Distal Implants on Bone Mechanics in Bar-retained Overdenture Treatment: A 3d Finite Element Study**

**as follows;**

The aim of this study is to evaluate, through static linear analysis using the three-dimensional finite element method, the stress generated in the bone surrounding implants in bar-retained four-implant overdenture designs, with a particular focus on the vertical level and distance discrepancy of the terminal implant. This study, which examines only specific scenarios, does not replicate all clinical conditions exactly. The planned prosthesis is an RP-4 design and does not receive support from soft tissues. Considering this, and to simplify the calculations, the prosthesis and mucosa were not modeled. The relevant loads were applied directly to the bar component.

The analyses were performed on a computer equipped with an Intel® Core™ i5-7500 processor, Nvidia GeForce GTX 1060 graphics card, 1 TB hard disk, and 16 GB RAM, running Windows 10 Pro and Ubuntu 20.04 operating systems. Blender 2.82 (Blender Foundation, community) was used to generate the three-dimensional geometry; Salome 9.8.0 (Électricité de France, Open Cascade, French Alternative Energies and Atomic Energy Commission) was used to build the three-dimensional solid model; and CalculiX 2.20 (Guido Dhondt, Klaus Wittig) was used for the finite element analysis.

The bone morphology was modeled in Blender based on a study concerning the morphology of the mandibular bone *(Barão VAR, Delben JA, Lima J, Cabral T, Assunção WG. Comparison of different designs of implant-retained overdentures and fixed full-arch implantsupported prosthesis on stress distribution in edentulous mandible – A computed tomography-based three-dimensional finite element analysis. J. Biomech. 2013 April; 46: 1312–1320.)*, including the edentulous mandible anterior to the mandibular third molar region. To minimize specific variables that could influence the outcome, the bone was modeled symmetrically relative to the sagittal plane, and no irregularities were introduced in the regions designated for implant placement. Subsequently, for each study group, the bone height in the area where the left terminal implant would be positioned was adjusted to the required dimensions. The cortical bone was modeled with an average thickness of 2 mm. The coordinates of the cross-sections of these models were transferred into the Salome software to generate the corresponding volumes.

The implants modeled in this study represent the screw-type implants that are most commonly preferred today. In the Salome software, the implants were created with a length of 10 mm and a diameter of 3.5 mm. To simplify the model, and because the stresses on the prosthetic components are outside the scope of this study, the abutment components were modeled as integrated with the bar and the implants (Figure 3.3). The abutment heights were selected to ensure a minimum clearance of 3 mm between the bar and the bone and to allow placement parallel to the occlusal plane. The bar was modeled with a diameter of 2 mm and a distal cantilever length of 8 mm.

Tetrahedral-element meshes were generated using the NETGEN algorithm in accordance with the model geometries. The elements on the implant surfaces were refined to a maximum size of 0.3 mm.

The mesh models were saved in UNV format within the Salome software and subsequently imported into CalculiX. Boundary conditions and material properties were defined in this environment, and the analysis was then carried out.

All degrees of freedom (DOF) in the posterior region of the bone were constrained to zero. Assuming complete osseointegration at the bone–implant interface, the interface elements were connected using the “bounded contact” option.

A vertical load of 100 N was applied to the left cantilever portion of the bar.

Analyses were performed across 28 configurations based on the vertical level discrepancy of the terminal implant and its distance from the mesial implant. The left terminal implant was examined at 1, 2, and 3 mm apical positions; 1, 2, and 3 mm occlusal positions; and at the same level as the other implants. The distance between the left terminal implant and its adjacent implant was modeled as 3.8, 4.7, 5.6, and 6.5 mm. The model visuals are presented below:

The results obtained from the analyses were transferred to Paraview (Kitware, Sandia National Laboratories, Los Alamos National Laboratory) for interpretation. In this study, von Mises stresses, as well as minimum and maximum principal stress and strain values, were visualized using color scales for each model.

Table: Material properties

|  | Young Modulus (Mpa) | Poisson's Ratio |
| --- | --- | --- |
| Cortical | 13700 | 0.30 |
| Trabecular | 1370 | 0.30 |
| Titanium (implant, abutment) | 110000 | 0.33 |
| Titanium grade 5 (bar) | 110000 | 0.28 |

A convergence analysis was done on one of the cases with maximum displacement values of 0.01215 mm 0.01204 mm and 0.01207 mm for maximum element sizes of 0.3, 0.5 and 0.7 mm respectively. Also maximum element size of 0.3 to 0.5 is standard in very similar studies (1-3).

1. Di Pietro N, Capparé P, Nagni M, et al. Finite element analysis (FEA) of the stress and strain distribution in Cone-Morse implant–abutment connection implants placed equicrestally and subcrestally. Appl Sci (Basel). 2023;13(14):8147. doi:10.3390/app13148147

2. Ceddia A, Assenza B, Bollero P, et al. Prediction of dental implant primary stability with cone beam computed tomography and finite element analysis. Materials (Basel). 2025;18(7):1625. doi:10.3390/ma18071625

3. Vautrin A, Bouchard P, Rieger D, et al. Homogenized finite element simulations can predict the mechanical response of bone-implant systems. J Mech Behav Biomed Mater. 2024;159:106158. doi:10.1016/j.jmbbm.2024.106158

**von Mises Stresses in the Cortical Bone**


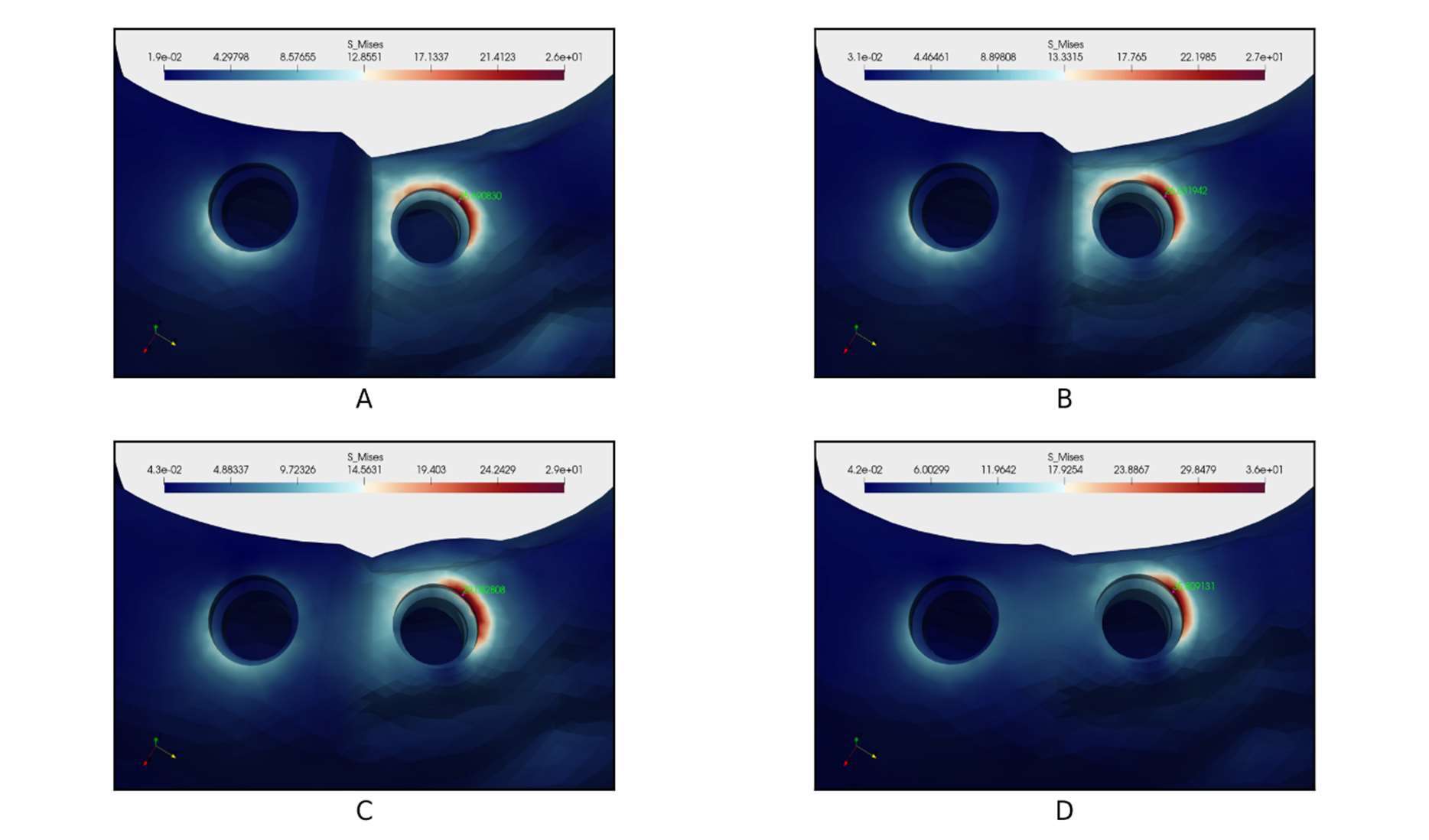

von Mises stresses in the cortical bone. In all models, the distance between the left terminal implant and the mesial implant is 3.8 mm. The variable among the models is the vertical level of the left terminal implant relative to the other implants. A) 3 mm apical. B) 2 mm apical. C) 1 mm apical. D) Same level. E) 1 mm coronal. F) 2 mm coronal. G) 3 mm coronal.

von Mises stresses in the cortical bone. In all models, the distance between the left terminal implant and the mesial implant is 4.7 mm. The variable among the models is the vertical level of the left terminal implant relative to the other implants. A) 3 mm apical. B) 2 mm apical. C) 1 mm apical. D) Same level. E) 1 mm coronal. F) 2 mm coronal. G) 3 mm coronal.

von Mises stresses in the cortical bone. In all models, the distance between the left terminal implant and the mesial implant is 5.6 mm. The variable among the models is the vertical level of the left terminal implant relative to the other implants. A) 3 mm apical. B) 2 mm apical. C) 1 mm apical. D) Same level. E) 1 mm coronal. F) 2 mm coronal. G) 3 mm coronal.

von Mises stresses in the cortical bone. In all models, the distance between the left terminal implant and the mesial implant is 6.5 mm. The variable among the models is the vertical level of the left terminal implant relative to the other implants. A) 3 mm apical. B) 2 mm apical. C) 1 mm apical. D) Same level. E) 1 mm coronal. F) 2 mm coronal. G) 3 mm coronal.


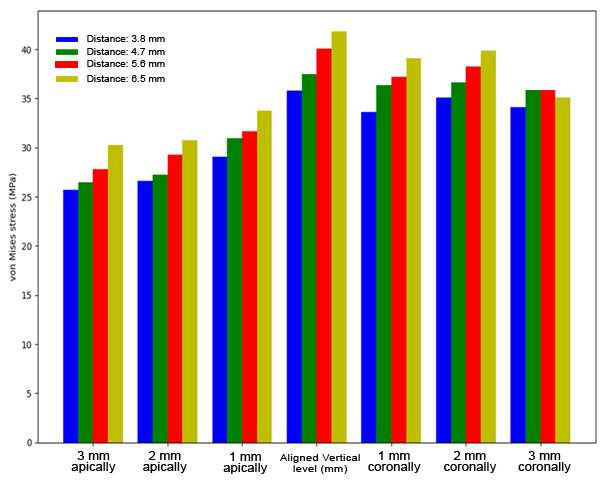


Maximum von Mises stresses in the study models.

**Tensile Stresses (Maximum Principal Stress) in the Cortical Bone**

Tensile stresses in the cortical bone. In all models, the distance between the left terminal implant and the mesial implant is 3.8 mm. The variable among the models is the vertical level of the left terminal implant relative to the other implants. A) 3 mm apical. B) 2 mm apical. C) 1 mm apical. D) Same level. E) 1 mm coronal. F) 2 mm coronal. G) 3 mm coronal.

Tensile stresses in the cortical bone. In all models, the distance between the left terminal implant and the mesial implant is 4.7 mm. The variable among the models is the vertical level of the left terminal implant relative to the other implants. A) 3 mm apical. B) 2 mm apical. C) 1 mm apical. D) Same level. E) 1 mm coronal. F) 2 mm coronal. G) 3 mm coronal.


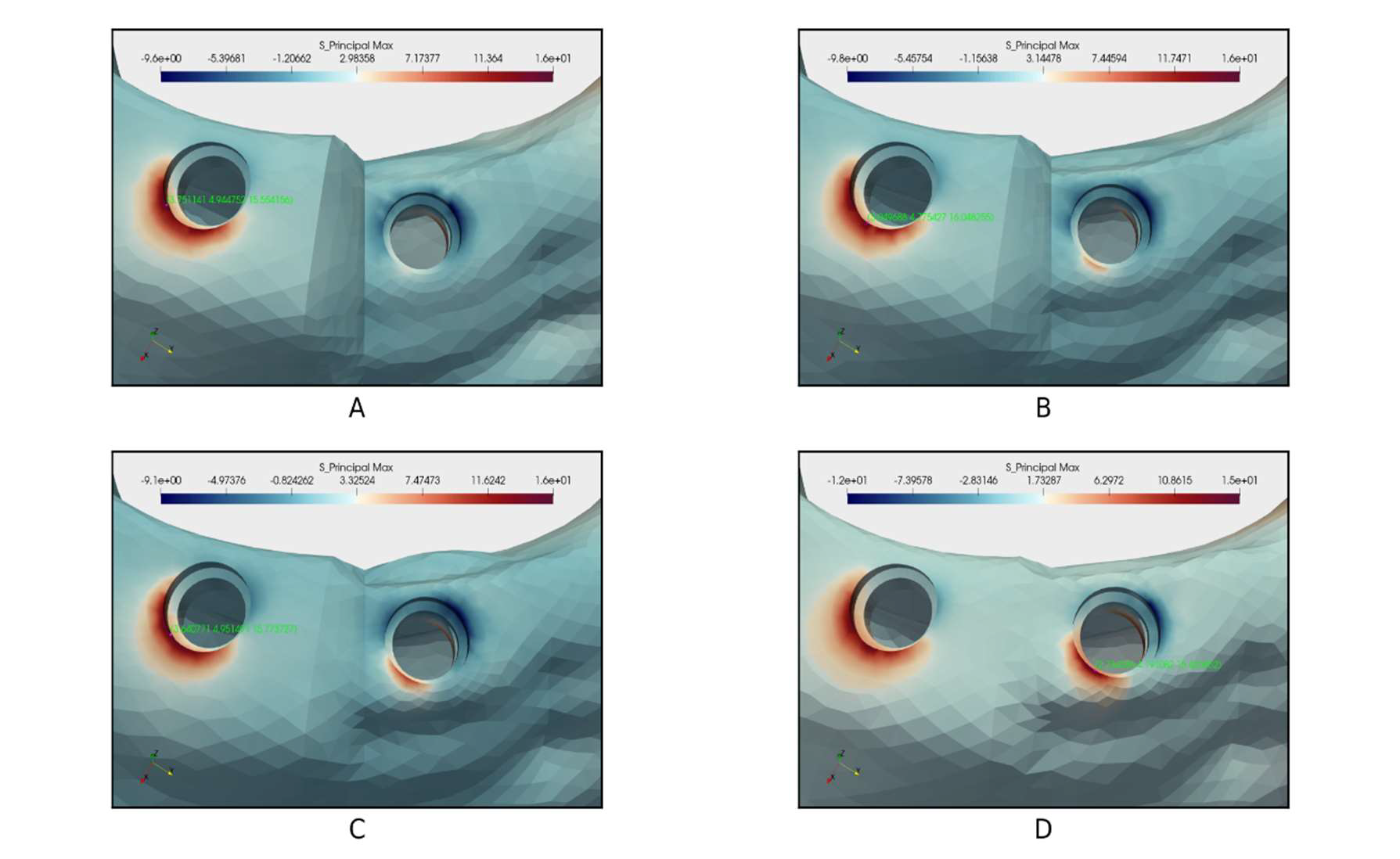

Tensile stresses in the cortical bone. In all models, the distance between the left terminal implant and the mesial implant is 5.6 mm. The variable among the models is the vertical level of the left terminal implant relative to the other implants. A) 3 mm apical. B) 2 mm apical. C) 1 mm apical. D) Same level. E) 1 mm coronal. F) 2 mm coronal. G) 3 mm coronal.

Tensile stresses in the cortical bone. In all models, the distance between the left terminal implant and the mesial implant is 6.5 mm. The variable among the models is the vertical level of the left terminal implant relative to the other implants. A) 3 mm apical. B) 2 mm apical. C) 1 mm apical. D) Same level. E) 1 mm coronal. F) 2 mm coronal. G) 3 mm coronal.


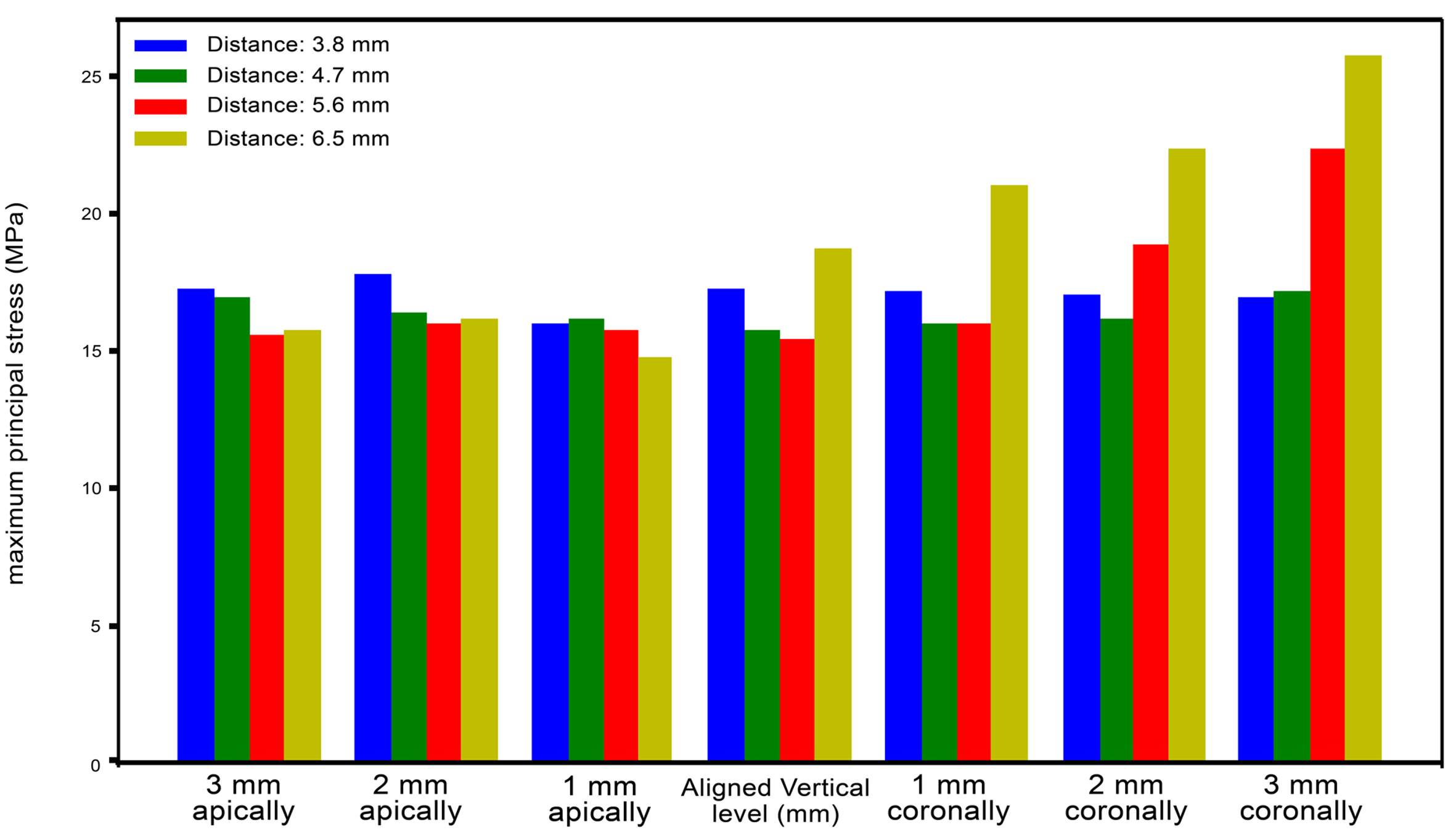


Maximum tensile stresses in the study models.

**Compressive Stresses (Minimum Principal Stress) in the Cortical Bone**

Compressive stresses in the cortical bone. In all models, the distance between the left terminal implant and the mesial implant is 3.8 mm. The variable among the models is the vertical level of the left terminal implant relative to the other implants. A) 3 mm apical. B) 2 mm apical. C) 1 mm apical. D) Same level. E) 1 mm coronal. F) 2 mm coronal. G) 3 mm coronal.

Compressive stresses in the cortical bone. In all models, the distance between the left terminal implant and the mesial implant is 4.7 mm. The variable among the models is the vertical level of the left terminal implant relative to the other implants. A) 3 mm apical. B) 2 mm apical. C) 1 mm apical. D) Same level. E) 1 mm coronal. F) 2 mm coronal. G) 3 mm coronal.

Compressive stresses in the cortical bone. In all models, the distance between the left terminal implant and the mesial implant is 5.6 mm. The variable among the models is the vertical level of the left terminal implant relative to the other implants. A) 3 mm apical. B) 2 mm apical. C) 1 mm apical. D) Same level. E) 1 mm coronal. F) 2 mm coronal. G) 3 mm coronal.

Compressive stresses in the cortical bone. In all models, the distance between the left terminal implant and the mesial implant is 6.5 mm. The variable among the models is the vertical level of the left terminal implant relative to the other implants. A) 3 mm apical. B) 2 mm apical. C) 1 mm apical. D) Same level. E) 1 mm coronal. F) 2 mm coronal. G) 3 mm coronal.


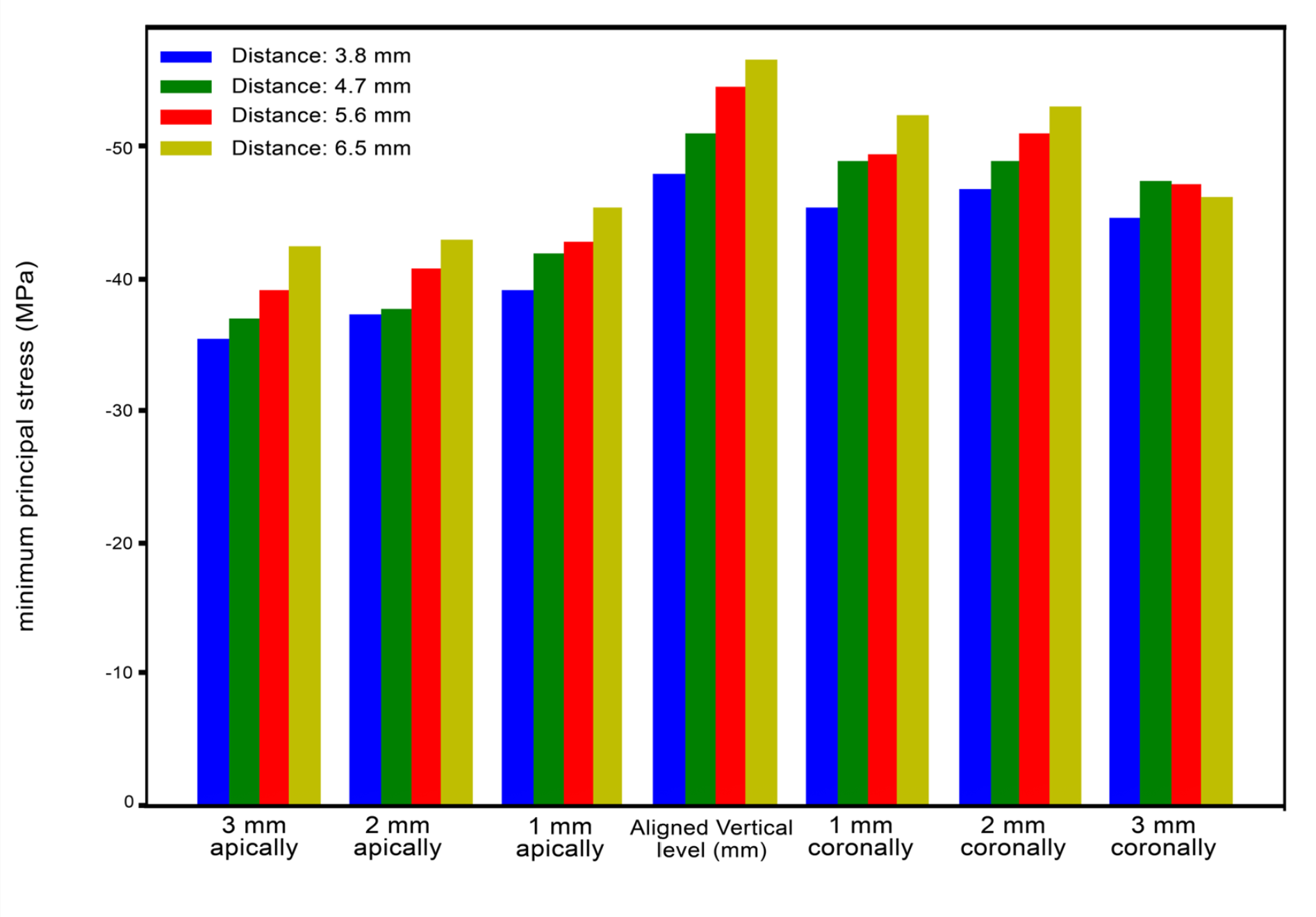


Compressive stresses in the study models.

**Tensile Strains (Maximum Principal Strain) in the Cortical Bone**

Tensile strains in the cortical bone. In all models, the distance between the left terminal implant and the mesial implant is 3.8 mm. The variable among the models is the vertical level of the left terminal implant relative to the other implants. A) 3 mm apical. B) 2 mm apical. C) 1 mm apical. D) Same level. E) 1 mm coronal. F) 2 mm coronal. G) 3 mm coronal.

Tensile strains in the cortical bone. In all models, the distance between the left terminal implant and the mesial implant is 4.7 mm. The variable among the models is the vertical level of the left terminal implant relative to the other implants. A) 3 mm apical. B) 2 mm apical. C) 1 mm apical. D) Same level. E) 1 mm coronal. F) 2 mm coronal. G) 3 mm coronal.

Tensile strains in the cortical bone. In all models, the distance between the left terminal implant and the mesial implant is 4.7 mm. The variable among the models is the vertical level of the left terminal implant relative to the other implants. A) 3 mm apical. B) 2 mm apical. C) 1 mm apical. D) Same level. E) 1 mm coronal. F) 2 mm coronal. G) 3 mm coronal.

Tensile strains in the cortical bone. In all models, the distance between the left terminal implant and the mesial implant is 6.5 mm. The variable among the models is the vertical level of the left terminal implant relative to the other implants. A) 3 mm apical. B) 2 mm apical. C) 1 mm apical. D) Same level. E) 1 mm coronal. F) 2 mm coronal. G) 3 mm coronal.


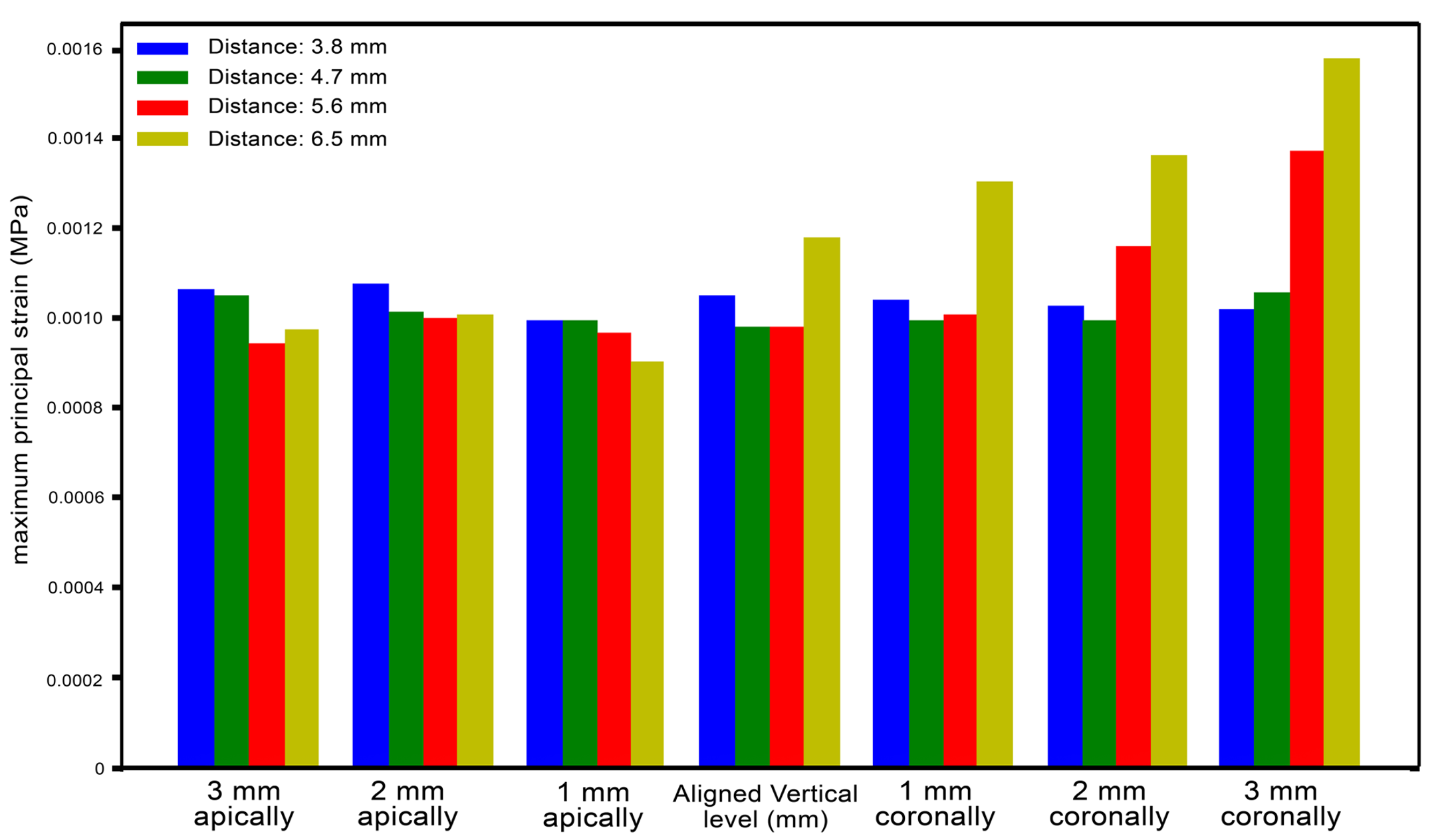


Maximum tensile strains in the study models.

**Compressive Strains (Minimum Principal Strain) in the Cortical Bone**

Compressive strains in the cortical bone. In all models, the distance between the left terminal implant and the mesial implant is 3.8 mm. The variable among the models is the vertical level of the left terminal implant relative to the other implants. A) 3 mm apical. B) 2 mm apical. C) 1 mm apical. D) Same level. E) 1 mm coronal. F) 2 mm coronal. G) 3 mm coronal.

Compressive strains in the cortical bone. In all models, the distance between the left terminal implant and the mesial implant is 4.7 mm. The variable among the models is the vertical level of the left terminal implant relative to the other implants. A) 3 mm apical. B) 2 mm apical. C) 1 mm apical. D) Same level. E) 1 mm coronal. F) 2 mm coronal. G) 3 mm coronal.

Compressive strains in the cortical bone. In all models, the distance between the left terminal implant and the mesial implant is 5.6 mm. The variable among the models is the vertical level of the left terminal implant relative to the other implants. A) 3 mm apical. B) 2 mm apical. C) 1 mm apical. D) Same level. E) 1 mm coronal. F) 2 mm coronal. G) 3 mm coronal.

Compressive strains in the cortical bone. In all models, the distance between the left terminal implant and the mesial implant is 6.5 mm. The variable among the models is the vertical level of the left terminal implant relative to the other implants. A) 3 mm apical. B) 2 mm apical. C) 1 mm apical. D) Same level. E) 1 mm coronal. F) 2 mm coronal. G) 3 mm coronal.


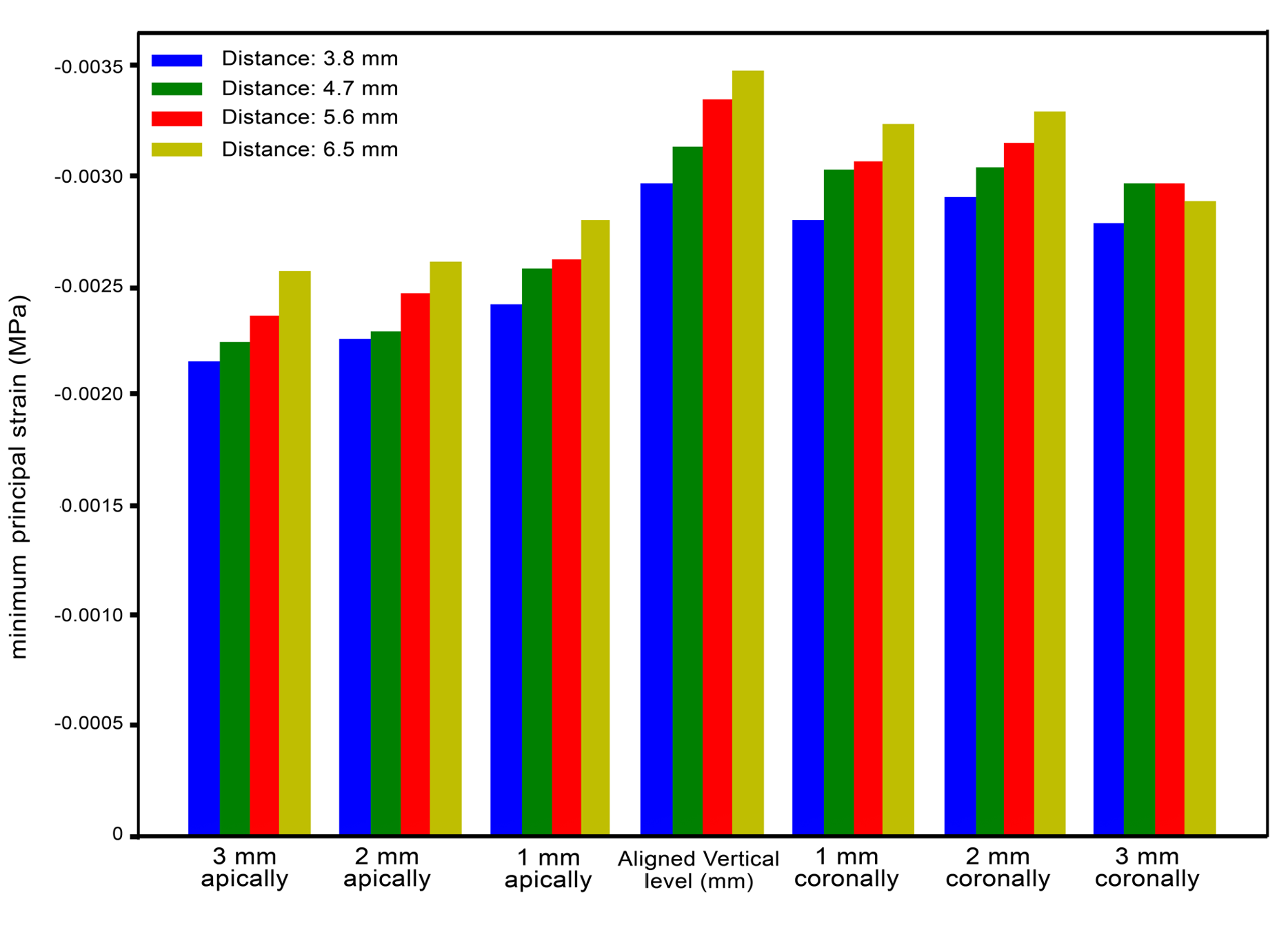


Compressive strains in the study models.
